# Inducible Liver Cancer Models in Transgenic Zebrafish to Investigate Cancer Biology

**DOI:** 10.3390/cancers13205148

**Published:** 2021-10-14

**Authors:** Ai Qi Lee, Yan Li, Zhiyuan Gong

**Affiliations:** Department of Biological Sciences, National University of Singapore, Singapore 119077, Singapore; aiqi@nus.edu.sg (A.Q.L.); dbsliya@nus.edu.sg (Y.L.)

**Keywords:** liver cancer, hepatocellular carcinoma (HCC), transgenic, sex disparity, tumour regression, cancer cachexia, metastasis, chemical screen, toxicology, zebrafish

## Abstract

**Simple Summary:**

Primary liver cancer ranks amongst the top ten cancers in frequency and mortality, with its global prevalence continuing to increase. Chemotherapy response remains poor and many therapies result in adverse side effects. The use of transgenic animal models that have high disease reproducibility provides opportunities to study cancer events that are difficult to study in human patients. Other than well-established mouse models in cancer research, now there is an increasing interest in using zebrafish to model various cancers. This review summarises the various oncogene transgenic zebrafish models for hepatocellular carcinoma (HCC) our laboratory has established, and the subsequent findings about liver cancer biology by using these transgenic models. In particular, most of our transgenic models employed chemically induced oncogene expression, allowing for temporal control and study of tumour initiation and regression. Investigation into these models reveals activation of tumorigenic pathways, inflammation, male-bias severity and skeletal muscle-wasting, characteristic of human HCC. Some of the models are also useful in screening for novel anti-metastatic drugs and evaluating the tumorigenic ability of environmental toxicants. The findings presented here demonstrate the utility of the zebrafish models for the further investigation of cancer biology and the development of novel and improved therapies.

**Abstract:**

Primary liver cancer is one of the most prevalent and deadly cancers, which incidence continues to increase while treatment response remains poor; thus, in-depth understanding of tumour events is necessary to develop more effective therapies. Animal models for liver cancer are powerful tools to reach this goal. Over the past decade, our laboratory has established multiple oncogene transgenic zebrafish lines that can be robustly induced to develop liver cancer. Histological, transcriptomic and molecular analyses validate the use of these transgenic zebrafish as experimental models for liver cancer. In this review, we provide a comprehensive summary of our findings with these inducible zebrafish liver cancer models in tumour initiation, oncogene addiction, tumour microenvironment, gender disparity, cancer cachexia, drug screening and others. Induced oncogene expression causes a rapid change of the tumour microenvironment such as inflammatory responses, increased vascularisation and rapid hepatic growth. In several models, histologically-proven carcinoma can be induced within one week of chemical inducer administration. Interestingly, the induced liver tumours show the ability to regress when the transgenic oncogene is suppressed by the withdrawal of the chemical inducer. Like human liver cancer, there is a strong bias of liver cancer severity in male zebrafish. After long-term tumour progression, liver cancer-bearing zebrafish also show symptoms of cancer cachexia such as muscle-wasting. In addition, the zebrafish models have been used to screen for anti-metastasis drugs as well as to evaluate environmental toxicants in carcinogenesis. These findings demonstrated that these inducible zebrafish liver cancer models provide rapid and convenient experimental tools for further investigation of fundamental cancer biology, with the potential for the discovery of new therapeutic approaches.

## 1. Introduction

Based on global cancer statistics in 2020, primary liver cancer was found to be the sixth most frequent type of cancer and ranked in third place for cancer mortality [1]. Its global incidence also continues to rise, with a study on liver cancer etiology showing that diagnosed cases have increased 114% from 1990 to 2016 [2]. For treatment of hepatocellular carcinoma (HCC), the most prevalent form of primary liver cancer, response to chemotherapy remains poor and the majority of therapies come with multiple adverse side effects [3,4]. The study and in-depth understanding of liver cancer initiation, progression and its related events is, hence, crucial for the discovery and development of preventive treatment and targeted cancer therapeutics with minimal side effects.

Animal models of liver cancer or HCC provide an invaluable way to study the disease. Since human liver cancers are asymptomatic at the early stages, presenting as other forms of liver disease instead [5], cancer is usually diagnosed only at later stages where cancer events such as tumour initiation and early progression cannot be studied; therefore, investigation of these events is largely dependent on using in vitro and experimental animal models. Activation and inhibition experiments, as well as drug screening, can also be performed easily in these models, allowing for the study of cancer events and the discovery of novel therapeutic drugs. Furthermore, different tissues and organs in the organism can be examined to study how liver tumours and liver cancer affect their function. In our laboratory, zebrafish are used as a model for liver tumours and HCC.

Due to its year-round availability, high fecundity, transparent embryos/larvae, and rapid development, the humble zebrafish has become an excellent vertebrate model for developmental and disease studies [6]. The advantages of utilising zebrafish as experimental models include, but are not limited to, the visualization of gene expression in a whole, intact organism via in situ hybridisation of embryos and high resolution live-imaging of fluorescence-tagged proteins or cells in a living vertebrate [7]. Robust genetic manipulation by microinjection of transposon and CRISPR/Cas9 systems into the one-cell embryos has allowed the generation of numerous transgenic genome-edited zebrafish lines [8].

Besides being used to study vertebrate development, to date, the zebrafish has been widely used to model numerous human diseases, including various cancers such as melanoma and leukemia [9,10]. These established models show significant phenotypic similarity with the human conditions. There is a surprising level of conservation across the two species despite the obvious evolutionary distance between zebrafish and humans. Barbazuk et al. discovered a high level of conserved synteny between the two species [11], while Howe et al. show that 71.4% of human genes have at least one obvious orthologue in the zebrafish, with orthologues for over 80% of human disease genes [12]. Several in-depth reviews on zebrafish as human disease models have been published [13,14,15], including one on using the Zebrafish Model Organism Database (ZFIN) to facilitate human disease research in zebrafish [16], and are cited here should the reader be interested in this topic. 

Many zebrafish models of liver cancer have been established in the past decade or so. The available transgenic models usually overexpress mutant forms of an oncogene or gene that is known to be involved in HCC, such as β-catenin [17], endothelin 1 [18], ubiquitin-like with PHD and RING finger domains 1 [19], and hepatitis virus proteins [20,21]. Clonal zebrafish lines have been established to allow for transplantable hepatic tumours, overcoming the previous issue of non-reproducibility of carcinogen-induced tumours [22]. More recently, several authors have also established xenograft tumour models of HCC using human HCC cell lines [23,24]. 

As comprehensive reviews of the various established liver cancer models in zebrafish have been recently published [25,26], this paper will not attempt to duplicate the effort. Instead, we will focus on the zebrafish liver cancer models that our lab has established in the past decade, our efforts to characterise and validate these models as well as novel findings about tumour initiation, progression and regression events from these models.

## 2. Beginning of Our Liver Cancer Journey

Our first foray into liver cancer started with the generation of carcinogen-induced hepatic tumours, which had previously been reported in mice [27,28] as well as in zebrafish [29,30,31]. Zebrafish were exposed to known carcinogens, 7,12-dimethylbenz(a)anthracene and dibenzo(a,l)pyrene, and were sampled 6–12 months after carcinogen exposure. Histological and DNA microarray analyses of these carcinogen-induced liver tumours showed that these zebrafish tumours were highly similar to human liver tumours not only at the histopathological level but also at the transcriptomic level as compared to other human tumour types. The transcriptomic data also showed the dysregulation of functional groups and molecular pathways which are seen to be frequently altered in human liver cancers. Moreover, the expression profiles of overlapping genes between human and zebrafish liver tumours correlated with tumour grade and progression [32,33]. The conservation between zebrafish and human liver tumours added to our confidence that the zebrafish could be used as a liver cancer model to study cancer-related events.

However, carcinogen-induced liver tumours generally take a long time (>6 months) to form. These tumours tended to be highly variable from one individual to another and are likely due to the alteration of different molecular pathways. In order to generate a more robust and reliable model to ensure reproducibility in studies, we adopted a genetic approach to establish transgenic zebrafish models for HCC by overexpression of selected oncogenes.

## 3. Established Transgenic Liver Cancer Models

### 3.1. Constitutive Oncogene Expression Model

**Figure 1:**
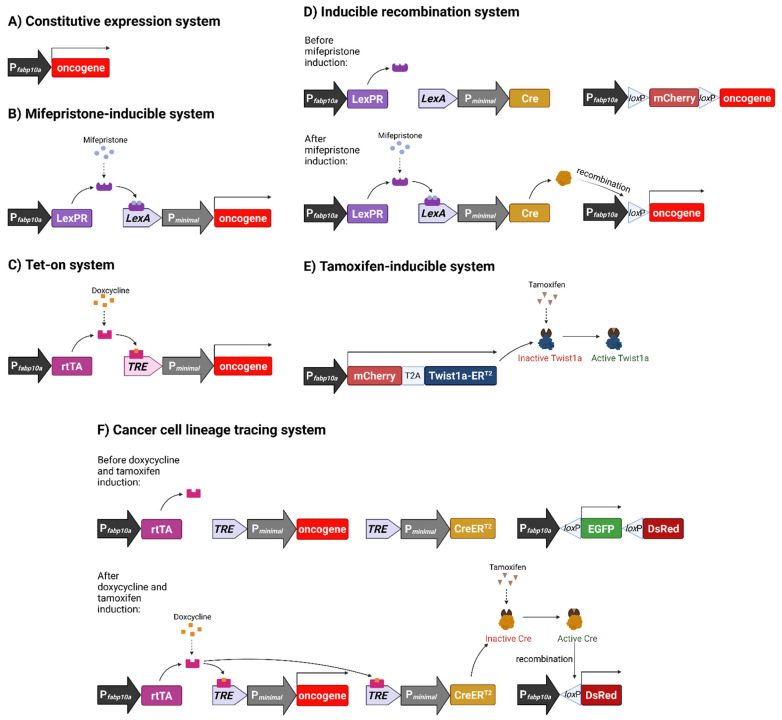
Schematic representation of various inducible systems we used for development of liver cancer models in transgenic zebrafish. (**A**) Constitutive expression system. Oncogene expression is under direct control of the hepatocyte-specific *fabp10a* promoter, causing oncogene to be constitutively expressed. (**B**) Mifepristone-inducible system. *fabp10a* promoter is used to transcribe a chimeric LexPR transactivator, which consists of the DNA binding domain of the bacterial LexA protein, the ligand binding domain of the human progesterone receptor (PR) and the activation domain of human p65 transcription factor. LexPR transactivator is activated by the mifepristone ligand and binds to the LexA operator to activate the transcription of the downstream oncogene. (**C**) Tet-on system. The reverse tetracycline-controlled transactivator (rtTA) is transcribed by the *fabp10a* promoter, but it becomes activated only in the presence of doxycycline and, in turn, it binds to the Tet Response Element (TRE) to activate the transcription of downstream oncogene. (**D**) Inducible recombination system. It incorporates the Cre-loxP components into the mifepristone-inducible system in order to induce permanent oncogene expression via genomic recombination. In this system, mifepristone activates LexPR, which binds the LexA operator to allow the expression of Cre recombinase. Cre recognises *loxP* sequences and excises the mCherry STOP cassette to permanently activate the downstream oncogene. (**E**) Tamoxifen-inducible system. A Twist1a-ER^T2^ fusion gene (*twist1a* and ligand binding domain of estrogen receptor [ER]) is constitutively transcribed by *fabp10a* promoter but the activation of the fusion protein requires the ligand, tamoxifen. T2A is a self-cleavage peptide to allow simultaneous production of two separate proteins. (**F**) Cancer cell lineage tracing system. Doxycycline activates rtTA, which in turn causes transcription of both *xmrk* oncogene and CreER^T2^. CreER^T2^ is activated by tamoxifen and excises the floxed EGFP sequence to cause the change of the expression of fluorescent protein genes. Figure created with BioRender.com (accessed on 23 September 2021).

Our first transgenic zebrafish liver cancer model constitutively expressed the hyperactive mutant oncogene *kras^V12^* under the control of the liver-specific promoter, *fabp10a* (Figure 1A) [34]. *KRAS* is part of the RAS/MAPK pathway, a signalling pathway that plays a prominent role in cell proliferation and differentiation. This oncogene has long been found to be mutated, and more recently, amplified, in various types of human cancers, such as colorectal and lung cancer [35,36,37,38,39,40]. Even though RAS mutations are rare in human HCC, RAS activation occurs despite the absence of its mutation [41]. Our transgenic model provided the opportunity to study the effect of liver-specific expression of oncogenic *kras^V12^* and its contribution to liver cancer in vivo as there was no mice model using *Kras* to drive liver tumorigenesis at that time.

Histological analysis of hepatic tumours confirmed the presence of hyperplasia, adenoma and HCC in the *kras^V12^* model [34]. The tumours also showed dysregulation of key signalling pathways seen in human HCC, such as MAPK and Wnt/β-catenin pathways, which were confirmed via molecular and transcriptomic analyses. Significant disease stage-specific conservation between the gene signatures of zebrafish and human liver tumours further validated the utility of the *kras^V12^* fish as a model for human liver cancer.

This model, however, has several drawbacks. First, early mortality due to heavy tumour burden made it difficult to study long-term cancer progression and to maintain the transgenic line as fish tended to die before sexual maturity. Secondly, constitutive oncogene expression starts from the earliest liver cell differentiation during embryonic development, which rarely happens in human liver cancers and makes it hard to study tumour initiation events. In order to overcome these limitations, we further developed several conditional transgenic models to have inducible oncogene expression in order to temporally control tumour initiation and progression. 

### 3.2. Chemically Inducible Oncogene and Cytokine Overexpression

Our first inducible model used the mifepristone-inducible LexPR system to control liver-specific *kras^V12^* expression (Figure 1B) [42,43]. This system allowed us to temporally control, and hence, study tumour initiation and regression; events that are difficult or even near impossible to study in human patients. Mifepristone-treated fish showed dosage and time-dependent tumour frequency and induction. At higher mifepristone concentrations, transgenic zebrafish livers turned hyperplastic after 1 week of induction and progressed to HCC within 4 weeks of treatment, with 100% penetrance. Western blots and immunohistochemistry confirmed the activation of the Raf-MEK-ERK pathway, while chemical inhibition of ERK and AKT pathways suppressed tumorigenesis in transgenic larvae [43].

Following the successful establishment and validation of the mifepristone-inducible model, our lab went on to generate additional transgenic lines by using another inducible system, the tetracycline-inducible Tet-on system [44], to control *kras^V12^* [45], *xmrk* [46], and mouse *Myc* [47] expression in the zebrafish liver (Figure 1C). Xmrk is a hyperactive mutant of fish epidermal growth factor receptor (Egfr) and structurally related to human EGFR [48], which plays a prominent role in human hepatocarcinogenesis and whose overexpression is correlated with poor survival [49]. Similarly, the amplification, and subsequent overexpression, of *MYC* is also associated with poor prognosis in human HCC [50] and has been shown to cause HCC in murine models [51,52]. All three transgenic lines showed rapid liver enlargement and tumorigenesis upon oncogene induction and the majority of tumours induced in the *kras^V12^* and *xmrk* lines progressed to HCC. The oncogenicity of zebrafish *myc* orthologs, *myca* and *mycb*, was also confirmed by transgenic expression, with *myca* transgenic zebrafish showing multinodular HCC after prolonged induction [53].

The utilisation of the two inducible transgenic expression systems has the advantage of selectively activating different oncogenes if multiple oncogenes are required in the study. The use of different chemical inducers also has an advantage in the case when any chemical inducer may interfere with downstream experimental analyses. In general, all these inducible oncogene transgenic zebrafish showed dosage- and time-dependent induction of oncogene expression and tumour severity. The induced tumour transformation is rapid and the HCC phenotype could be induced within 1–2 weeks of chemical induction [54]. The temporal induction could be performed at any age such as from 2 weeks old to 8 months old to reliably induce HCC phenotype [46]. In larvae (3–7 days postfertilisation), rapid oncogenic liver growth can be observed immediately after chemical induction and 2D liver size can be quantitatively measured for various bioassays before feeding. The tumour transformation is generally homogenous throughout the liver, which is an advantage for biochemical analyses but is unlikely to be the case in human patients where the cancer originates from one or a few transformed cells and progresses through clonal expansion. 

Besides the described oncogene-driven liver cancer models, we have also established a mifepristone-inducible zebrafish line that overexpresses *tgfβ1a* specifically in the liver [55]. The cytokine is of interest because its expression is upregulated in diet-induced non-alcoholic steatohepatitis (NASH) and it might play an important role in the transformation of NASH to HCC. Indeed we found that chronic Tgfβ1 overexpression induced both HCC and cholangiocarcinoma (CCA), bile duct epithelial cell cancer, with dose-dependent severity. It appears that there was a switch of dominant activated signalling pathways from Smad to Erk during carcinogenesis in hepatocytes while CCA requires concurrent activation of both Smad and Erk pathways [55].

### 3.3. Inducible Permanent Oncogene Expression

In all of the above mentioned inducible transgenic models, the fish had to have constant exposure to the inducing chemical in order to maintain transgene expression. This resulted in the transformation of all liver cells once a chemical inducer is used, which is unlikely to be the case in human cancer. Furthermore, the continued presence of the chemical inducer may also affect subsequent chemical treatment and this is not desired in many experiments, such as drug screening, using these models. To overcome these problems, we developed another transgenic model which requires only a short pulse chemical treatment to activate oncogene expression by induction of permanent genomic change. In this model, we combined the mifepristone-inducible *kras^V12^* model and the Cre/loxP system (Figure 1D) [56]. Like in the previous model, *kras^V12^* was controlled by the liver-specific promoter *fabp10a*, but its transcription is interrupted by *loxP* sequences flanking an *mCherry*-STOP cassette sequence in the new transgenic line. Cre recombinase expression, induced in the presence of mifepristone, excises the STOP cassette sequence, resulting in genomic recombination and the permanent expression of *kras^V12^*. In practice, it was found that Cre-mediated recombination tended to be incomplete. This resulted in distinct tumour nodules within the liver that did not show any mCherry fluorescence, suggesting that nodules were likely formed from clonal proliferation, which more resembled human liver tumours [56].

### 3.4. Metastatic Model of HCC

So far, in all transgenic models we generated by overexpression of a single oncogene, although histologically proven HCC is robustly induced, there was no obvious tumour metastasis. One likely reason is the high severity of the induced primary liver cancer; as it occurs in the whole liver, and these fish may not be able to survive till metastasis stage. Another reason is that tumour metastasis may require the activation of additional molecular pathways. To tackle this issue, we generated another transgenic zebrafish line with mifepristone-induced expression of a gene (*twist1a*) important in epithelial-mesenchyme transition (EMT), the first step of tumour metastasis. The new *Twist1a-ER^T2^* line constitutively expressed liver-specific Twist1a-ER^T2^, but activation of the protein only occurs upon tamoxifen treatment (Figure 1E) [57]. When we combined the *Twist1a-ER^T2^* and *xmrk* transgenic fish, we found that hepatic cells in double transgenic fish showed extensive dissemination from the tumorigenic liver and even long-distance migration to trunk and tail regions [57]. In some fish, the establishment of secondary tumours was also observed [58]. These observations suggested the need for cooperation of oncogene and EMT gene to initiate tumour metastasis in the zebrafish models.

A summary of our established transgenic zebrafish models of liver cancer is presented in Table 1.

### 3.5. Histological, Transcriptomic and Molecular Validation

All of our established models have been extensively validated histologically. In general, zebrafish, rodent, and human liver neoplasms are very similar in histology [32]. Though part of the HCC grading criteria of human HCC does not entirely fit for zebrafish, nuclear factors seen in the disease in both species are highly alike. The most severe tumour grades in humans and zebrafish show the most pronounced nuclear irregularity, hyperchromasia, and obvious nucleoli [32].

Molecularly, liver tumours from several of our transgenic models have been subjected to transcriptomic analyses and cross species comparison. In the microarray analysis of our first non-inducible *kras^V12^* model, we observed the activation of several important signalling pathways that are also seen in human liver hyperplasia and HCC. Cross-species analysis also showed that upregulated zebrafish hyperplastic liver-enriched genes and HCC-enriched genes were significantly associated with human dysplastic liver and HCC respectively. A few genes from the gene signatures that the analyses surfaced had also been reported as prognostic markers in human HCC [34]. RNA-sequencing and cross species analyses of HCC samples from *xmrk* fish showed a significant resemblance to human HCC S2 subtype that was categorised by a meta-analysis of nine patient cohorts across the globe [63]. Myc and its co-activator MAX were among the top 10 most dysregulated transcription factors in the *xmrk* liver, further validating this model as the S2 subtype is also characterised by the activation of a Myc signature [64]. For the *Myc* transgenic model, cross-species transcriptome analyses revealed the highest correlation to Myc transgenic mouse compared to other mice models of HCC. The zebrafish tumours also showed significant similarity with human HCC as compared to lower-grade tumours—a result that was consistent even when two different human HCC datasets were used. Moreover, 16 Myc target genes, retrieved from the Molecular Signatures Database [65], were found to be amongst the upregulated genes from the zebrafish Myc tumours. The 16-gene signature was able to predict Myc transgenic mice and differentiate between lower and higher-grade human liver cancers [47]. Taken together, these findings strongly suggest the conservation of molecular pathways in Myc-induced liver tumours across the three species. In a transcriptome study of all three transgenic liver tumours, induced by *kras^V12^*, *xmrk* and *Myc*, all three types apparently showed the highest similarity with very advanced human HCC as compared to various human liver disease conditions from cirrhotic nodules to various grades of HCC [66]. Moreover, the common up- and down-regulated genes found from these three zebrafish tumour models also showed the same up- and down-regulation in human HCC, suggesting the conservation of fundamental genes necessary for neoplasia.

Along with studying the histology and transcriptome of liver tumours, we also investigated the dysregulation of multiple signalling pathways that have been implicated in oncogene-signalling and tumorigenesis. In *kras^V12^*-expressing liver tumours, progressively increasing activation of MAPK and Wnt/β-catenin signalling pathways was observed from benign to malignant tumours [34,56]; inhibition of Wnt-signalling also reduced oncogenic liver size in *Myc* fish [67]. These two pathways are prominently activated in human liver cancer [68,69,70]. The loss of p53 function in human liver cancer is one of the most frequent mutations [71,72] and shows poor prognosis [73]; its loss in *kras^V12^* and zebrafish *myca* lines also results in accelerated tumour progression and mortality rates [34,54]. Inhibition of the VEGF/FGF pathway suppressed liver enlargement in induced *kras^V12^* and *Myc* fish, implicating that VEGF/FGF signalling and angiogenesis is necessary for oncogenic liver tumour development in zebrafish [67]. Hypervascularity is not only commonly found in human HCC, but many treatments for advanced HCC also target angiogenesis, such as the well-known multikinase inhibitor, sorafenib, which inhibits angiogenesis via the VEGF pathway [74,75]. The crossing of our *Myc* and *xmrk* lines resulted in quicker and more severe liver carcinogenesis than *Myc* or *xmrk* single transgenic fish. Interestingly, the Warburg effect, or aerobic glycolysis, is potentially at play in double transgenic tumours. The *Myc*/*xmrk* tumours might have a preference for acquiring energy via glycolytic pathways as transcriptome analysis revealed a downregulation of fatty acid and amino acid metabolism genes and upregulation of critical glycolytic genes such as *pkm2* [62], one of the rate-limiting enzymes in aerobic glycolysis [76]. These characteristics are also seen in transcriptomic and metabolomic studies on human HCC [77,78,79]. These dysregulated molecular pathways and their outcomes seen in the induced zebrafish liver tumours are also observed in human liver cancer, implying that liver tumour events in zebrafish likely occur via similar pathways as those in humans.

## 4. Important Findings from Our Models

### 4.1. Oncogene Addiction: Tumour Regression and Re-Induction

Spontaneous regression of tumours is rare in humans, and is usually associated with the stimulation of the immune system of cancer patients [80]. One of the most intriguing observations from our inducible transgenic zebrafish models is that the induced tumours show the ability to regress when the oncogene is no longer expressed by the withdrawal of the chemical inducer. The tumours can be re-induced by exposing the fish to the inducer again to re-activate oncogene expression. Since our models show that the expression of a single oncogene is sufficient to induce liver tumours and HCC, these observations altogether indicate the presence of an oncogene addiction phenomenon, where cancer initiation and maintenance is dependent on a single or a few oncogenes [81]. The regressed tumours in our models shrink in size and revert to normal liver histology [43,46,54]. Transcriptomic analysis also revealed a reversal of molecular changes in regressing tumours compared to tumours with progressing HCC [64].

There are two possible mechanisms of tumour regression: (1) tumour hepatocytes undergo cell death and are eliminated while normal hepatocytes eventually replace these tumour hepatocytes via proliferation and differentiation, and (2) tumour hepatocytes might be directly reverted to normal hepatocytes. In order to test the two possibilities, we developed a *CreER* transgenic line that allowed us to trace liver cell lineage (Figure 1F) [82]. In this transgenic line, tamoxifen-activated Cre protein, CreER^T2^, is expressed only in the presence of doxycycline. It also includes liver-specific promoter-controlled EGFP flanked by *loxP* sequences, and a *DsRed* gene at the end. When crossed with the *xmrk* line, the double transgenic *CreER/xmrk* fish express EGFP in all hepatocytes, which start to express *xmrk* and progress into liver tumours upon doxycycline induction. The addition of tamoxifen initiates Cre/loxP recombination where existing tumour hepatocytes begin to permanently express DsRed instead of EGFP. This allowed for the distinction between DsRed-expressing hepatocytes from tumour-cell lineage and newly differentiated EGFP-expressing hepatocytes after tamoxifen is removed. Upon initiation of tumour regression by cessation of doxycycline treatment, we observed a large increase in apoptosis and a decrease in proliferation of liver tumours within the first week. After four weeks of tumour regression, we observed both DsRed- and EGFP-expressing hepatocytes in normal liver. Histological and transcriptomic analyses confirmed that both populations of hepatocytes are normal when compared to wildtype control hepatocytes. Hence, our studies demonstrated the possibility of reversion of tumour cells to normal cells by suppression of the major addictive oncogene or oncogenic pathway [82].

### 4.2. Immune Response and Inflammation

It is known that a wide range of innate and adaptive immune responses are involved in the development and progression of human liver cancer, having both tumour-promoting and anti-tumour capabilities [83,84,85]. Investigation into the microenvironment of the induced liver tumours has shown that the immune system is very much involved in zebrafish liver cancer as well.

It is clear that neutrophils and macrophages play a tumorigenic role in our transgenic zebrafish models. With the help of fluorescence-labelled neutrophils and macrophages in several transgenic zebrafish strains [86,87], we were able to study innate immune cell infiltration and localisation as well as their involvement in liver tumour initiation and progression. Neutrophils are rapidly recruited to the *kras^V12^* larvae liver upon induction, with a significant increase in neutrophil density in the liver from 8-16 hours post-induction (hpi) onwards [88,89]. Time-lapse confocal microscopy revealed that neutrophils move actively around the *kras^V12^* liver but remain relatively stationary after infiltration, in contrast to the control liver, where neutrophils were equally active regardless of location. *kras^V12^* liver only became significantly enlarged from 24 hpi, suggesting the tumorigenic function of neutrophils in the liver [88,89]. An increase in tumour-associated neutrophils (TANs) after oncogene induction was also seen in *Myc* fish [90], and both neutrophil and macrophage numbers in the liver increased upon *xmrk* induction [64]. The inhibition of neutrophils and macrophages was able to attenuate tumour growth in *kras^V12^* larvae [59], while neutrophil depletion and inhibition of neutrophil differentiation in *Myc* fish also suppressed tumour growth [90]. These observations indicate that neutrophils and macrophages have pro-tumour functions in tumorigenesis, but their involvement in HCC regression has likewise been noted. Like in the HCC liver, TAN and tumour-associated macrophage (TAM) numbers are increased in the early and late regressing *xmrk* tumours. The immune cells seem to be clustered regionally, with TAMs clearly clustering around blood vessels, compared to their uniform distribution in the HCC liver. Neutrophils and macrophages likely have different functions in tumorigenesis and tumour regression [64].

How neutrophils and macrophages are involved in zebrafish liver cancer is complex due to crosstalk in the tumour microenvironment. Cause-and-effect relationships are not only one-way, but might be bi-directional, suggesting the presence of feedback loops. On one hand, angiogenesis seems to aid neutrophil recruitment to the hepatic *Myc* tumour and tumorigenesis as inhibition of angiogenesis decreases TAN numbers and tumour growth [90]. On the other hand, a decrease in TANs lowers tumour–blood vessel density [91]. Neutrophils and macrophages were also found to contribute to hepatic stellate cell (HSC) activation, with macrophages also supporting HSC survival. HSCs in turn secrete Tgfβ1 which promotes TAN and TAM tumorigenic function [89].

Further investigation suggests that certain inflammatory cytokines also contribute to hepatocarcinogenesis either directly or by inducing immune cells. *kras^V12^* hepatocytes increase expression of *tgfβ1a*, likely recruiting and inducing neutrophils, which promote a pro-inflammatory tumorigenic microenvironment. Tgf-β depletion and inhibition decreased liver size and neutrophil density, and partially rescued TAN anti-tumour cytokine expression [88]. This has been further confirmed in another study where tgf-β signalling inhibition also reduced liver size and downregulated expression of pro-tumour genes in hepatocytes, TANs and TAMs [89]. In our liver-specific *tgfβ1a* model, *tgfβ1a* overexpression progressively induced liver inflammation during tumour initiation [92]. An investigation into cholangiocyte involvement in HCC indicated that *il17a/f1* expression was significantly upregulated and secreted by cholangiocytes, in turn directly augmenting *kras^V12^* liver carcinogenesis. Il17a/f1 inhibition reduced liver growth and TAN and TAM numbers [93].

### 4.3. Sex Disparity and Hormones

Gender disparity in human primary liver cancer has been well-documented, with liver cancer being 2 to 4 times more prevalent in males than in females [94,95]. Differential gene expression and pathway activation between the two sexes has been observed in human patients [96]. Likewise, this phenomenon has also been observed in our transgenic zebrafish models and has been investigated to further elucidate mechanisms in HCC-related sex disparity.

HCC develops and progresses faster in male *kras^V12^* fish than in female *kras^V12^* fish [59,60]. After 10 days of doxycycline induction, all male liver tumours showed HCC, with 80% at an advanced stage, while only 70% of females had early HCC. Male tumours were also more severe after long-term induction, exhibiting higher proliferation, Wnt/β-catenin pathway activation, and loss of E-cadherin, an important tumour-suppressing molecule. Male tumours advanced to have multiple nodules suggestive of different tumour colonies, while their female counterparts remained homogenous. Complete regression of male tumours also took a longer time [60]. The male-bias phenomenon is also seen in *xmrk*, *Myc*, and *Myc*/*xmrk* transgenic fish [61,97]. *Xmrk* and *myc*/*xmrk* males developed higher grade HCC quicker than females and male tumours from all three lines showed a higher rate of proliferation. *Myc*/*xmrk* double transgenic tumour regression was also delayed in male fish.

Sex hormones have been found to contribute to the processes of hepatic tumorigenesis and tumour regression in zebrafish. Androgen treatment in both male and female *kras^V12^* fish stimulated cell proliferation in the liver tumour, accelerating tumour growth and progression. The opposite was seen in estrogen-treated fish [60]. Similar observations were made in *Myc*/*xmrk* fish, where androgen-treated females developed HCC faster and regressed slower than their doxycycline-only controls, while estrogen-treated males showed opposite effects [97]. To further investigate the role of androgen in HCC, or more specifically, androgen-receptor signalling, a liver-specific androgen receptor knockout line (L-ARKO) was developed, validated, and crossed with the *kras^V12^* line. The absence of androgen receptors indeed attenuated tumour development and cell proliferation during early tumorigenesis. Interestingly, all fish went on to develop HCC, indicating that the tumorigenic role of androgen-receptor signalling is restricted to the early stages of tumour development [98].

Male and female fish show differences in tumour microenvironment as well. Male *kras^V12^*, *xmrk*, and *Myc* tumours recruit more neutrophils and macrophages than corresponding female tumours [54,59], an observation confirmed in human pre-HCC and HCC samples [59]. TANs and TAMs from males also show greater pro-tumour gene expression than immune cells from the female tumour. Cortisol, an adrenal hormone, was more robustly produced in the male liver and contributed to the increased production of tgfβ1 [59], which has been mentioned earlier to possess pro-tumour functions in tumorigenesis. In another study, it was found that serotonin expression and production was higher in the *kras^V12^* male tumour, enhancing HSC numbers and activation. The stimulated HSCs then contribute to the increase in tgfβ1 production [61]. Higher serotonin levels, tgfβ1 production, and HSC numbers were also observed in *xmrk* and *Myc* male tumours [54]. More importantly, increases in cortisol, serotonin and tgfβ1 levels have been verified in human samples [61,90]. It is interesting to note that some non-sex hormones such as cortisol and serotonin are also involved in sex disparity of liver tumour formation.

**Figure 2:**
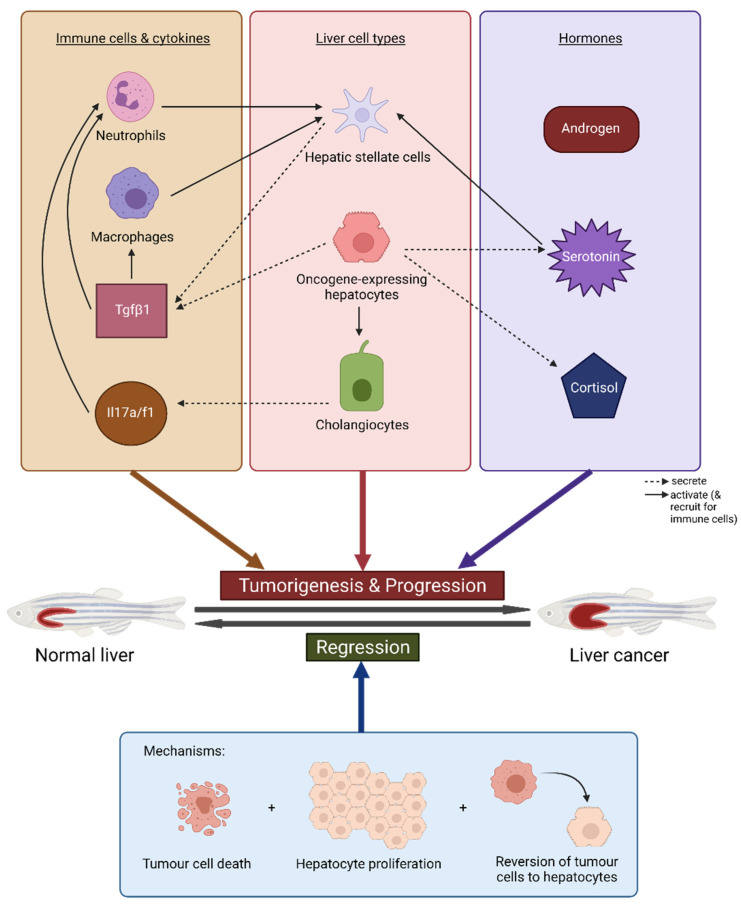
Summary of key pathways and mechanisms involved in tumour development and regression in our transgenic zebrafish models of liver cancer. The figure displays the main liver cells (hepatocytes and cholangiocytes) and immune cell types, cytokines and hormones that contribute to or exacerbate tumorigenesis and progression to liver cancer, and the crosstalk between them. The three mechanisms for tumour regression are also shown. Figure created with BioRender.com (accessed on 17 August 2021).

The key pathways and mechanisms involved in tumour development and regression are summarised and illustrated in Figure 2.

### 4.4. Muscle-Wasting and Cancer Cachexia

Cancer-associated cachexia is a syndrome whose main symptoms are the loss of skeletal muscle mass and adipose tissue, amongst others. Though mechanisms and mediators of cancer cachexia have surfaced in the past few years, there is currently still no effective medical treatment for cachexia [99,100]. In the treatment of human cancers, although nutritional interventions had no impact on survival rates [101], starvation therapy has been proposed as a novel treatment that could also improve response to current therapies [102,103]. To further investigate muscle-wasting accompanied with cancer progression, the zebrafish model could be an excellent experimental system for mechanistic investigation as the nutrition effect can be easily manipulated and investigated in this model organism.

Examination of our induced *kras^V12^* fish indeed revealed progressive wasting of the skeletal muscles [104]. Fish body weight and muscle fibres decreased significantly as doxycycline treatment progressed. Underfeeding was able to slow down carcinogenesis but the rate of muscle-wasting was increased due to insufficient nutrition. However, overfeeding led to both faster carcinogenesis and faster muscle-wasting, which was more severe than the muscle-wasting seen in underfed fish, strongly suggesting that HCC was heavily involved in the muscle-wasting phenomenon. Leptin, a hormone that regulates energy homeostasis [105], was identified to be involved in HCC-induced muscle-wasting as well as in human liver disease. Expression of leptin was significantly increased in *kras^V12^* livers compared to wildtype control fish, an increase that was further exacerbated by overfeeding. Human liver samples also displayed a significant increase in leptin expression as liver disease progressed, which was the highest in HCC samples. To investigate the role of leptin in HCC-induced muscle-wasting, a leptin-receptor (Lepr) knockout line was generated and crossed with *kras^V12^* fish. After tumour induction, Lepr mutant *kras^V12^* fish had better survival rates and significantly reduced muscle-wasting in all differentially fed groups, though carcinogenesis remained unaffected. Treatment of Lepr mutant *kras^V12^* fish with a myostatin inhibitor further reduced muscle-wasting and loss of body weight. These observations show that leptin signalling indeed plays a critical role in HCC-induced muscle-wasting and a combination of leptin signalling and myostatin inhibition might prove to be an effective treatment strategy for cancer cachexia patients [106].

### 4.5. Chemical and Toxicological Screening Using Zebrafish HCC Models

A huge advantage that the zebrafish model has over rodents is the ability to do high-throughput in vivo chemical screening, such as for novel drug discovery or preclinical drug screening [106,107]. To explore the feasibility of using our oncogene transgenic zebrafish larvae for potential chemical screens, we treated *kras^V12^* larvae with known pathway inhibitors and were able to grade drug efficacy according to liver sizes following treatment with different chemicals [43]. Results indicated that ERK and AKT pathways both need to be inhibited for the total suppression of tumorigenesis. Since the liver is labeled by fluorescent protein expression in most of our oncogene transgenic zebrafish models, we further refined the protocol by quantitative measurement of 2D liver size in zebrafish larvae using online available ImageJ software and this might be developed into a potentially high-throughput and automated chemical screening platform [67,108]. We also used *Twist1a-ER^T2^*/*xmrk* fish to carry out a small scale chemical screening for anti-metastasis drugs. We started with 67 FDA-approved drugs and found 3 known human drugs that were able to reduce the frequency of fish with dissemination and migration of tumour cells from the liver. Further validation analyses identified adrenosterone as the drug that could suppress the dissemination of highly metastatic human cancer cell lines in zebrafish xenotransplants by inhibition of the novel target, HSD11β1 [57].

Though the drugs for cancer treatment identified in chemical screens using our zebrafish models have yet to be tested in clinical trials, many other laboratories have found some potential drug compounds based on zebrafish screens and several of these compounds have already entered clinical trials [109,110]. For example, North et al. performed a chemical screen in zebrafish and identified prostaglandin E_2_ (PGE_2_) to be capable of stimulating haematopoietic stem cells (HSCs) [111]; 16,16-dimethyl-PGE_2_ (dmPGE_2_), a stable derivative of PGE_2_, showed the ability to enhance HSC proliferation, survival, and homing to bone marrow, as well as long-term stable engraftment in transplantation models [112]. The compound subsequently entered phase I clinical trials for use in umbilical cord blood (UCB) treatment for transplantation in leukemic patients, who were co-transplanted with one treated UCB and one untreated UCB. Results showed sustained engraftment and long-term hematopoiesis as a result of the dmPGE_2_-treated UCB unit in the majority of patients [113]. A phase II trial also revealed a lower incidence rate of graft vs. host disease [109]. Mandelbaum et al. have developed a cultured zebrafish pluripotent blastomere system to screen for inhibitors of the *MYB* gene, which is overexpressed in adenoid cystic carcinoma (ACC). A retinoic acid agonist, all-trans retinoic acid (ATRA), was found to decrease gene expression in the zebrafish blastomeres and could suppress tumour growth in patient-derived xenograft models [114]. Early reports on an ongoing phase II clinical trial of ATRA suggest stabilisation in advanced ACC patients [110].

Besides being utilised for drug screening, the oncogene transgenic zebrafish model could also be used to screen and assess how various chemicals affect tumorigenesis. This approach is particularly valuable to test environmental toxicants for their potential impact in inducing or promoting carcinogenesis. We have tested the *kras^V12^* fish with several different categories of environmental toxicants such as heavy metals and organic toxicants for their effects on inflammation and liver tumorigenesis based on neutrophil count and liver size respectively. Increases in inflammation were accompanied by corresponding increases in tumorigenesis, once again showing the important role inflammation plays in tumour formation [115]. The *kras^V12^* fish model including adult fish has also been used by other laboratories to evaluate the effects of environmental toxicants, tris(1,3-dichloro-2-propyl) phosphate (TDCIPP) and perfluorooctane sulfonate (PFOS). Exposure to these two chemicals caused more severe HCC and increased HCC frequency, respectively [116,117,118].

## 5. Concluding Remarks

As global liver cancer prevalence continues to increase and treatment response remains poor, the development of new and improved therapies is highly desired. Over the past decade, our lab has developed several conditional transgenic systems and established chemically inducible transgenic zebrafish lines that have shown to be robust models of HCC and its related processes such as metastasis and muscle-wasting. Using these models, we have elucidated various molecular insights into the different mechanisms involved in liver cancer initiation, progression and regression. Chemical screening using the fish has also revealed novel anti-metastatic drugs as well as the inflammatory and carcinogenic ability of certain environmental toxicants. These observations and findings provide potential options to consider in the discovery and development of new therapies to treat liver cancer. Furthermore, our successful strategies may also be applicable for the development of cancer models in other organs/tissues.

So far, cancer cachexia studies in fish is only limited to muscle wasting. In human cancer patients, cachexia can also present with adipose tissue-wasting, inflammation and alterations in smell and taste and gastrointestinal tract hormone levels [119]. A rat HCC model presenting weight and skeletal muscle loss also showed a progressive decline in cardiac muscle mass and cardiac function [120]. Future works can focus on the characterisation of HCC’s effects on other organs in fish, which could further allow for the screening of more treatment options.

In a study, we also demonstrated the feasibility for transformed cancer cells to be reverted to normal cells after the suppression of expression of the addicted oncogene. This observation provides a therapeutic basis for using a chemical drug to target the addicted oncogene. We also observed that cancer-reverted cells were more susceptible to re-induction of carcinogenesis and thus our model may also provide a model to investigate cancer dormancy and recurrence. We have used a transgenic recombination approach to trace cancer cell lineage during tumour regression, a more powerful multi-color lineage tracing approach followed by single cell RNA-seq technology could be further applied to identify potentially different classes of cancer-reverted cells.

The development of early diagnosis markers for HCC has been of high importance, as patients are usually diagnosed at advanced stages and the well-studied serum marker α-fetoprotein has limited sensitivity and specificity. With the chemically-inducible liver tumour models, we are able to study tumour initiation and progression at different stages. Making use of proteomic and metabolomic approaches, our transgenic models could be used to explore new serological biomarkers for HCC. Since the zebrafish liver tumour models also displayed gender disparity, biomarker exploration can be performed in a gender-specific manner.

## Figures and Tables

**Table 1:** Summary of our transgenic zebrafish models of liver cancer.

Transgene	Transposon System	Inducible System	Permanent Recombination	Tumour Phenotypes *	References
*kras^V12^*	Ac/Ds	-	-	3 mpf: 36% HP 6 mpf: 22% HA 9 mpf: 26% HCC	[34]
	Ac/Ds	LexPR	No	1 wpi: 100% HP 4 wpi: 100% HCC	[43]
	None	Tet-on	No	7 dpi: 40% HCC and 50–60% HA in males. 30–40% HA and 60–70% HP in females. 10 dpi: 100% HCC in males. 70% HCC, 20% HA and 10% HP in females. 5mpi: 100% multinodular HCC in males, 100% homogenous HCC in females.	[45,59,60,61]
	Ac/Ds	LexPR	Yes	Severity dependent on age of induction. Most fish developed mosaic livers with more than 1 tumour type.	[56]
*xmrk*	None	Tet-on	No	7 dpi: 50% HCC, 30% HA and 20% HP in males. 20% HA and 80% HP in females. 6 wpi: 100% HCC	[46,54,62]
Mouse *Myc*	None	Tet-on	No	3 wpi: 30% HA, 70% HP 16 wpi: 7 out of 8 showed HA, 1 showed HCC	[47]
Zebrafish *myca* and *mycb*	Ac/Ds	LexPR	No	Liver tumour progression from HP, HA to multinodular HCC	[53]
*tgfβ1a*	Ac/Ds	LexPR	No	6 wpi: 30% mixed HCC + CCA, 20% HCC + BHP, 10% HCC, 20% HA, 20% HP + BHP	[55]
*Twist1a-ER^T2^/xmrk*	Ac/Ds	Tet-on	No	8 dpi: 46% showed distant dissemination of tumour cells, 39% showed abdominal dissemination and the rest showed no dissemination	[57]

HP—liver hyperplasia, HA—hepatic adenoma, HCC—hepatocellular carcinoma, CCA—cholangiocarcinoma, BHP—bile duct cell hyperplasia, mpf—months post fertilisation; wpi—weeks post induction; dpi—days post induction; mpi—months post induction. * Transgenic lines showed dosage- and time-dependent tumour incidence and severity.
